# Ranking Hits From Saturation Transfer Difference Nuclear Magnetic Resonance–Based Fragment Screening

**DOI:** 10.3389/fchem.2019.00215

**Published:** 2019-04-12

**Authors:** Jonas Aretz, Christoph Rademacher

**Affiliations:** ^1^Department of Biomolecular Systems, Max Planck Institute of Colloids and Interfaces, Potsdam, Germany; ^2^Department of Biology, Chemistry, and Pharmacy, Freie Universität Berlin, Berlin, Germany

**Keywords:** fragment-based drug discovery, fragment-based drug design, saturation transfer difference nuclear magnetic resonance spectroscopy, STD NMR, screening

## Abstract

Fragment-based screening is an established route to identify low-molecular-weight molecules to generate high-affinity inhibitors in drug discovery. The affinities of these early hits from fragment screenings require a highly sensitive biophysical screening technique. Saturation transfer difference (STD) nuclear magnetic resonance (NMR) is one of the most popular methods owing to its high sensitivity for low-affinity ligands. It would be highly beneficial if rank-ordering of hits according to their affinity from an initial or counter-screen could be performed—a selection criterion found in the literature. We applied Complete Relaxation and Conformational Exchange Matrix (CORCEMA) theory adapted for saturation transfer (ST) measurements (CORCEMA-ST) calculations to predict STD NMR results from a large set of fragment/receptor pairs to investigate the boundaries under which the assumption holds true that a high STD effect can be applied to select for higher-affinity fragments. Overall, we come to the conclusion that this assumption is invalid.

## Introduction

Fragment-based drug design (FBDD) has successfully complemented the toolbox for developing small-molecule pharmaceuticals (Baker, 2012; Hann and Keserü, 2012) as highlighted by Vemurafenib and Venetoclax, the first drugs entering the market originating from FBDD (Bollag et al., 2010; Souers et al., 2013). In this approach, fragments of drug-like molecules ranging between ~150 and 250 Da in size are identified binding to a receptor. These initial hits are then developed into high-affinity leads following fragment evolution strategies such as fragment linking, fragment growing, or fragment merging (Rees et al., 2004).

The screening of fragment collections is challenged by the low affinities of the initial hits, which typically range between dissociation constants of 10 μM and 10 mM. These values are target dependent, but the average fragment hit resides in the higher micromolar to the single-digit millimolar range. To overcome the low affinity of fragments, sensitive biophysical techniques are commonly employed such as surface plasmon resonance (SPR), X-ray crystallography, and techniques from nuclear magnetic resonance (NMR). In particular, ligand-observed NMR techniques such as STD NMR are the most frequently used methods owing to their high sensitivity and low false-positive rate (Gossert and Jahnke, 2016).

During an STD NMR experiment, receptor resonances are selectively saturated for a given time (saturation time, *t*_sat_) by a series of frequency-specific pulses. This magnetization spreads in milliseconds in the hydrogen network within the receptor *via* spin diffusion (Mayer and Meyer, 1999; Jayalakshmi and Krishna, 2002). Furthermore, the magnetization is transferred to low-molecular-weight ligands, enabling the identification of actives from compound mixtures (Mayer and Meyer, 1999; Jayalakshmi and Krishna, 2002). The ligand will then dissociate from the receptor site and saturated ligands accumulate free in solution, which results in a decreased signal intensity of the bulk ligand. This spectrum is subtracted from a reference spectrum of the same sample recorded in the absence of saturation. Hence, signals in an STD spectrum correspond to ligands that bound to the receptor. Moreover, saturation transfer to the ligand is distance dependent and ligand hydrogens receiving more saturation are considered in close proximity to the receptor interface in the bound state (Mayer and Meyer, 2001). A binding epitope can thus be derived, normalizing the saturation transfer to the proton receiving the highest saturation. Additionally, the magnitude of saturation transfer is affected by the affinity and the kinetics of complex formation (Jayalakshmi and Krishna, 2002; Meyer and Peters, 2003). Finally, ligand as well as receptor saturation is counteracted by nuclear relaxation processes, particularly T_1_ relaxation, leading to a dissipation of the magnetization to the bulk solvent. Consequently, the saturation build-up of ligand equilibrates at longer duration of the saturation time (Jayalakshmi and Krishna, 2002).

The coupled dipolar relaxation network of receptor and ligand hydrogens can be calculated using the complete relaxation and conformational exchange matrix (CORCEMA) theory. With this formalism, STD NMR experiments can be simulated for a given receptor/ligand complex, and CORCEMA-ST has been successfully applied to refine such complexes (Jayalakshmi and Krishna, 2002, 2005; Szczepina et al., 2011). Moreover, CORCEMA calculations allow one to reduce the complexity of the STD NMR experiment in theory to explore parameters influencing the saturation transfer. For example, two receptor/ligand complexes can be compared assuming that they share exactly the same affinity and, by that, rule out effects arising from the exchange kinetics. This then allows extracting the influence of the geometry of the binding site (Jayalakshmi and Krishna, 2002). Previous CORCEMA calculations using a single receptor/ligand pair indicated a correlation between affinity and saturation transfer to the ligand (Jayalakshmi and Krishna, 2002). Sufficient residence time of the ligand in the binding site allows transfer of the magnetization. Consequently, saturated ligand molecules accumulate free in solution and the overall signal intensity of the corresponding ligand resonances is decreased. When the affinity is exceeding a certain threshold, the release of ligand from the receptor site is limited and the STD effect decreases again. Taken together, a bell-shaped plot of affinity vs. saturation transfer is expected.

Here, we calculate theoretical STD effects over a broad range of receptor–ligand pairs. These insights are combined with experimental results from STD NMR screening and fragment-based ligand design. Next, we investigated whether rank-ordering of fragment-sized ligands from primary screening data based on the STD amplification factors is suitable. Overall, evidence from calculations as well as experimental data suggests that such rank-ordering is invalid.

## Materials and Methods

### Structure Preparation

Fragment/protein complexes were selected from the Protein Data Bank (PDB) database based on resolution and diversity of the proteins and ligands, and avoiding sterical clashes between the ligand and protein originating from unreasonably low distances. All complexes were prepared in Molecular Operating Environment (MOE, version 2015; Chemical Computing Group ULC., 2018). Hydrogens were added at pH 7; if necessary, missing loops were introduced followed by a structure refinement step as implemented in MOE using standard parameters and manual inspection. Complexes and their respective affinities are given in Table S1 in the order they appear throughout the study.

### CORCEMA-ST

CORCEMA-ST (version 3.8) was run on a regular desktop computer (Jayalakshmi and Krishna, 2002). If not stated otherwise, the following parameters were assumed: ligand concentration [L] = 1 mM; protein concentration [P] = 20 μM; *k*_on_ = 10^9^ M^−1^ s^−1^; saturation times *t*_sat_ = 0.5, 2.0, and 8.0 s; correlation time of the ligand τ_c_ = 5 × 10^10^ s. Exchange-mediated leakage is not taken into account in this model when more than one binding site is assumed. To significantly decrease the calculation time, only hydrogens in an 11-Å radius surrounding the ligand were included (Figure S5). Methyl resonances of amino acids were assumed to be instantaneously saturated during the simulation. Only non-exchangeable hydrogens of the receptor and the ligand were included in the calculation, and all other atoms were removed from the input structures. Only protons with the highest saturation transfer were used for the analysis.

### Saturation Transfer Difference Nuclear Magnetic Resonance Measurements

**Figure 1:**
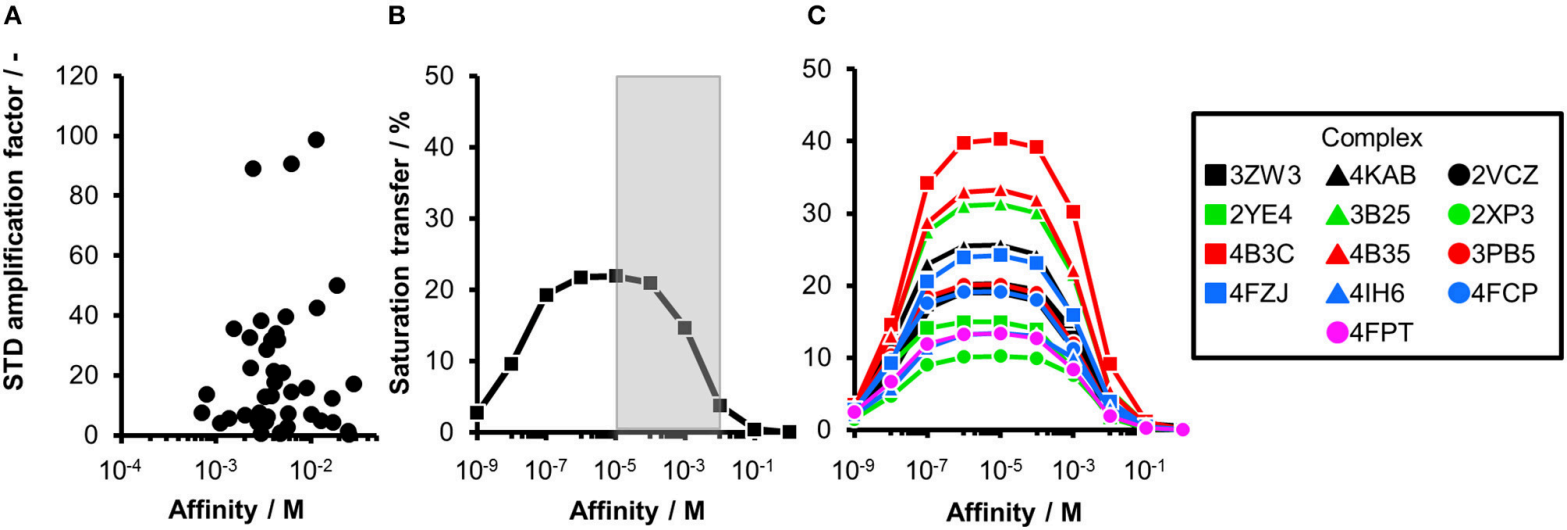
Comparing experimental and calculated STD effects using CORCEMA-ST. **(A)** STD amplification factors observed during fragment screening of langerin with [P] = 10 μM, [L] = 200 μM, and *t*_sat_ = 4 s plotted against affinity estimated by SPR (Aretz et al., 2018). **(B)** Thirteen fragment/protein complexes were used to perform CORCEMA-ST calculations of the saturation transfer from the receptor to the low-molecular-weight ligand. The average over all complexes is shown and depicts the saturation transfer to the ligand proton receiving the highest saturation. A gray box highlights the affinity regime typically populated by fragments (*K*_d_ ranging from 10 μM to 10 mM). **(C)** Individual plots of all 13 complexes highlighting the high dispersion of saturation transfer. For CORCEMA-ST calculations, typical STD NMR screening conditions were assumed: [P] = 20 μM, [L] = 1.0 mM, saturation time = 2.0 s, τ_c, bound_ = 30 ns (corresponding to 50 kDa molecular weight), and *k*_on_ = 10^9^ M^−1^ s^−1^.

**Figure 2:**
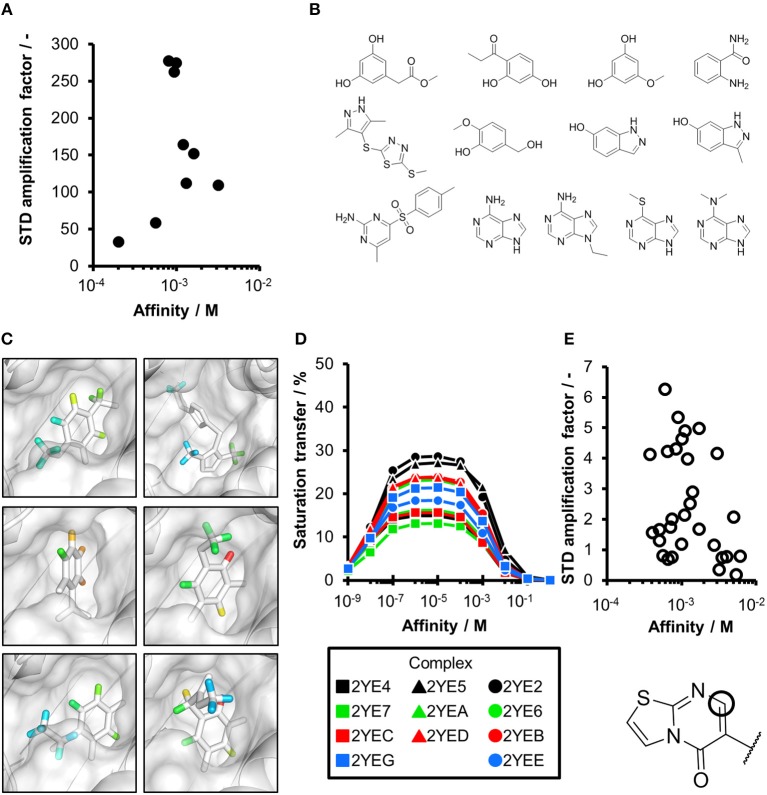
CORCEMA-ST calculations and experimental data of fragments binding to the same binding site. **(A)** STD amplification factors determined with CORCEMA-ST of fragments binding to PDE10A and AmpC. For these fragments, a crystal structure and thermodynamic data were available (Barelier et al., 2014; Recht et al., 2014). **(B)** Chemical structures of the fragments used to analyze HSP90 (Roughley and Hubbard, 2011) and **(C)** examples of some of these ligands in complex with protons colored by their normalized STD effect (red being the highest STD effect and blue being the lowest effect). **(D)** Saturation transfer with varying dissociation constants is shown for all complexes shown in **(B)** for HSP90. For all complexes, the same parameters were assumed: [P] = 20 μM, [L] = 1.0 mM, τ_c, bound_, = 30 ns, and *k*_on =_ 10^9^ M^−1^ s^−1^. **(E)** STD amplification factors derived from a structure-activity relationship (SAR) study of thiazolopyrimidine derivatives binding to langerin plotted against affinity determined by SPR (Aretz et al., 2018). STD NMR measurements were performed with [P] = 20 μM, [L] = 500–1,000 μM, depending on compound solubility, and *t*_sat_ = 1 s, considering only the proton in the 7-position (carbon highlighted with a circle).

STD NMR experiments were performed as described elsewhere (Aretz et al., 2018). Briefly, 10 μM murine langerin was screened against a library of 660 fragments at a ligand concentration [L] = 0.2 mM using a saturation time of *t*_sat_ = 4 s. Hits were counter-screened by SPR (see the section Estimation of Affinity Using Surface Plasmon Resonance), enabling affinity estimation and hit validation. For SPR-validated hits, the STD amplification factor was determined as reported earlier (Mayer and Meyer, 2001) and plotted against affinity (Figure 1A). For epitope mapping of thiazolopyrimidine derivatives (Figure 2E), protein concentration was [P] = 20 μM, ligand concentration was [L] = 0.5–1 mM, and *t*_sat_ = 1 s. Affinity of all derivatives was estimated by SPR (see the section Estimation of Affinity Using Surface Plasmon Resonance), and the STD amplification factor was determined as reported earlier for the proton in the 7-position of the thiazolopyrimidine scaffold (Mayer and Meyer, 2001). Spectra were analyzed in MestReNova 10.0.0. Compound structures, estimated affinities, and STD amplification factors are given in Table S2.

### Estimation of Affinity Using Surface Plasmon Resonance

SPR experiments were performed on a Biacore T100 (GE Healthcare) as described elsewhere (Aretz et al., 2018). Briefly, murine langerin was immobilized on a CM7 Series S chip to a density of 7,500 RU using NHS/EDC coupling. Subsequently, dilution series of compounds in 25 mM HEPES, pH 7.6, 150 mM sodium chloride, 5 mM calcium chloride, and 0.005% Tween-20 buffer were injected with a flow rate of 10 μl min^−1^.

## Results and Discussion

Ideally, a fragment screening technique enables rank-ordering of hits according to their affinity. Due to recent reports on rank-ordering hits from STD NMR screening and counter-screening applying the STD amplification factor (Jose et al., 2012; Begley et al., 2013; Cala and Krimm, 2015), we retrospectively analyzed a fragment screening against langerin, a C-type lectin receptor involved in pathogen uptake by immune cells (Aretz et al., 2018). Here, fragments were screened by STD NMR, and the affinity of hits was subsequently estimated by SPR (Figure 1A). Although the affinity of all hits was in a suitable range for STD NMR, there was no correlation between STD amplification factor and affinity (Pearson's correlation coefficient). Consequently, rank-ordering of hits according to their STD amplification factor would have been misleading in this example.

To analyze the missing correlation between STD amplification factor and affinity more systematically, we assembled a diverse set of receptor/fragment complexes (Table 1) and computed the outcome of an STD NMR experiment using CORCEMA-ST (Figure 1B). Setting a molecular weight of the receptor to a typical drug target of 50 kDa, e.g., checkpoint kinase 1 (CHK1), we evaluated the influence of the affinity on the saturation transfer. This setup simulates the situation during STD NMR-based fragment screening with a larger panel of different fragments being present in the same mixture potentially occupying multiple sites with varying geometry and physicochemical properties. The calculated saturation transfer plotted against the affinity averaged over all complexes resulted in a bell-shaped curve as reported earlier (Figure 1B; Jayalakshmi and Krishna, 2002). For the typical affinity range of fragments, which is between 10 μM and 10 mM, these plots indicate elevated saturation transfer with increasing affinity (gray box, Figure 1B), suggesting that rank-ordering hits from primary screening would be suitable. However, the analysis of the individual contributions of the fragment complexes to the average depiction revealed a high variability of the calculated saturation transfer (Figure 1C), demonstrating that even assuming the same thermodynamics and kinetics, a high variability of the STD NMR readout is to be expected. This trend was also observed for saturation times of 0.5 and 8.0 s (Figure S1). Overall, this variability suggests that binders from initial STD NMR screening cannot be rank-ordered assuming that the magnitude of the STD effect would correlate with fragment affinity.

**Table 1:** Protein–ligand complexes used for CORCEMA-ST calculations.

**PDB ID**	**Name (long)**	**Name (short)**	**Protein class**	**Ligand**
2XP3	Peptidyl-prolyl cis-trans isomerase NIMA-interacting 1 protein	Pin1	Isomerase	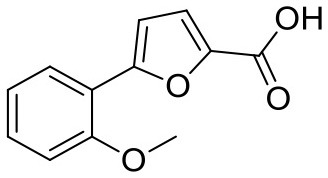
2YE4	Heat shock protein 90	HSP90	Chaperone	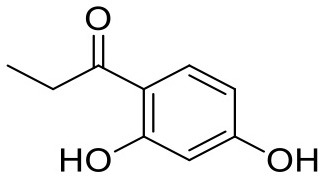
3B25	Heat shock protein 90	HSP90	Chaperone	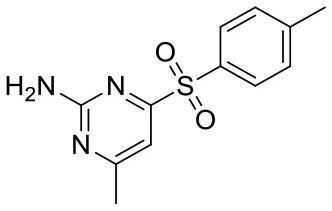
3PB5	Endothiapepsin	EAPA	Protease	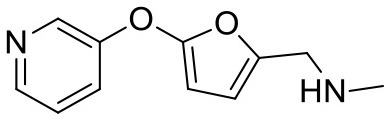
4B3C	DNA repair and recombinant protein RADA	RadA	ATPase	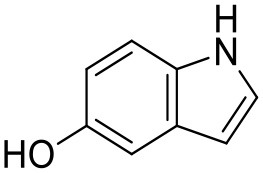
4B35	DNA repair and recombinant protein RADA	RadA	ATPase	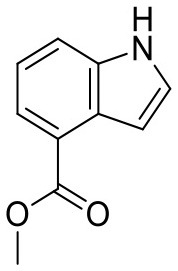
4FCP	Heat shock protein 90	HSP90	Chaperone	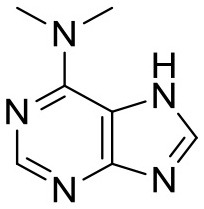
4FZJ	Pantothenate synthetase	Pts	Ligase	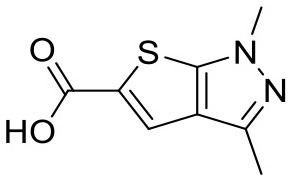
4IH6	HCV non-structural protein 5B	NS5B	RNA Pol.	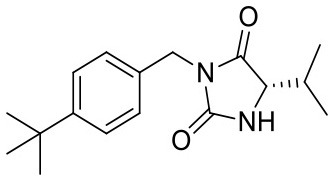
4FPT	Carbonic anhydrase 2	CA2	Lyase	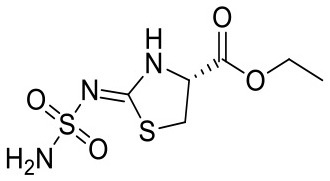
2VCZ	Prostaglandin D2 synthase	PGDS	Isomerase	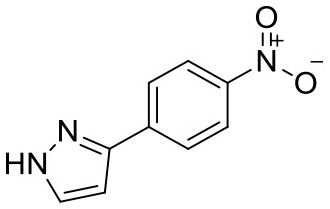
3ZW3	Phosphatidylinositol-4,5-bisphosphate 3-kinase	PI3K	Kinase	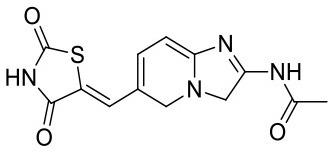
4KAB	Focal adhesion kinase 1	FAK1	Kinase	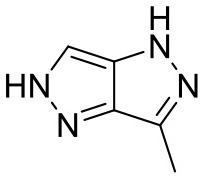

To ensure that a fragment is binding to the targeted site during the STD NMR screening process, typically competition experiments are employed. Consequently, under these conditions, the saturation transfer for all fragments to be rank-ordered originates from the same binding site. Hence, to eliminate the effect of the composition of the binding site, a series of fragments binding to a single receptor pocket was utilized using two exemplary drug targets: phosphodiesterase 10A (PDE10A) and beta-lactamase (AmpC). In both cases, crystallographic and thermodynamic data were previously reported. From these reports, kinetic data were calculated assuming diffusion-controlled on-rate kinetics and hence STD amplification factors were determined (Figure 2A; Barelier et al., 2014; Recht et al., 2014). Similar to our experimental screening data, there was no correlation between calculated STD amplification factor and affinity (Pearson's correlation coefficient, *p* > 0.05). We then focused on another receptor with a high availability of fragment-bound crystal structures, i.e., heat shock protein 90 (HSP90), to rule out effects coming from the binding site geometry (Figures 2B–D; Roughley and Hubbard, 2011). A more homogeneous saturation transfer profile was observed (Figure 2D) in comparison to the simulated screening data with multiple binding sites (Figure 1C). Still, fragments were indistinguishable based on their affinity (Figure 2D). Taken together, if binding to a single protein pocket can be assumed, slight chemical variations in the structure of the fragments, which are typically found in a series of derivatives, lead to substantial variability of the observed STD effects.

To experimentally validate this finding, we analyzed STD data of a structure-activity relationship study (SAR) of thiazolopyrimidine derivatives binding to langerin (Figure 2E; Aretz et al., 2018). These derivatives only differ in substitutions of the 6-position; hence, the STD amplification factor of the same aromatic proton in the 7-position was determined. Under these conditions, a linear correlation between affinity and STD amplification factor can be inferred (Pearson's correlation coefficient, *p* < 0.05), which is in contrast to the screening results. Still, the compound with the highest affinity ranked 19 of 30; thus, the high variability of the STD effect predicted by CORCEMA is in agreement with these experimental data.

**Figure 3:**
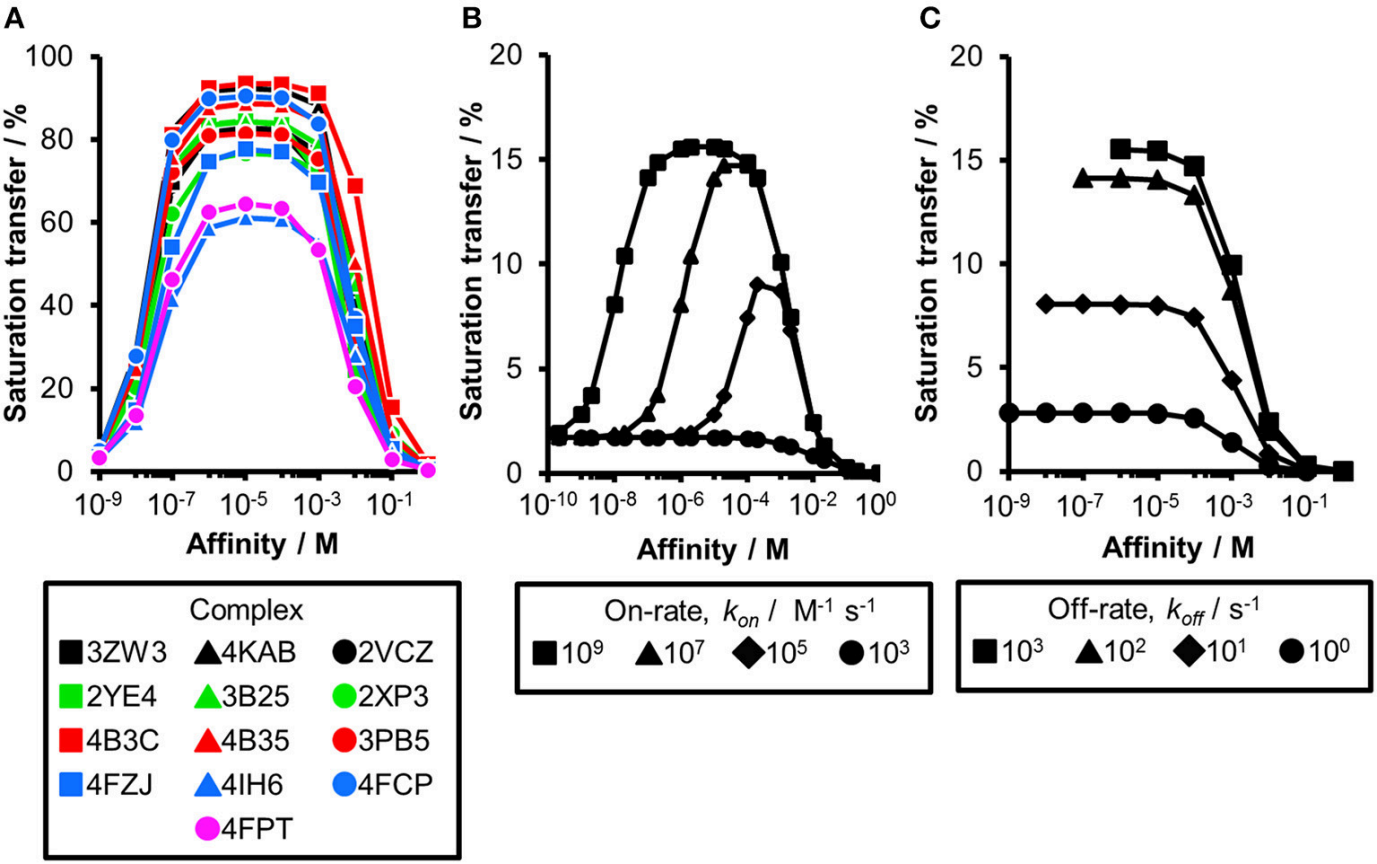
The influence of altered kinetics and molecular weight of a receptor on the STD amplification factor. **(A)** CORCEMA-ST calculations were performed for 13 fragment/protein complexes assuming a molecular weight of 166 kDa. CORCEMA-ST calculations were performed on the same fragment/protein complexes as above (Figure 1; Table 1), but changing the protein correlation time to 100 ns [τ_c, bound_ = 100 ns, [P] = 20 μM, [L] = 1.0 mM, *k*_on =_ 10^9^ M^−1^ s^−1^]. **(B,C)** CORCEMA-ST calculations for CA2 varying on- and off-rates of the receptor/ligand complex. Saturation times of 8.0 s are shown [PDB ID: 4FPT, [P] = 20 μM, [L] = 1.0 mM].

Thus far, our analysis was focused on receptors with a molecular weight of 50 kDa. As the saturation transfer to the ligand is increased with the increase in molecular weight, we hypothesized that increasing the correlation time τ_c_ to 100 ns in our calculations might compensate for the difference in chemical composition of the binding sites. Such decrease in molecular tumbling rate corresponds to a ~166 kDa receptor. While the CORCEMA calculations predicted elevated STD effects and a more homogeneous saturation transfer compared to our results at 50 kDa (Figure 1B), these data still do not allow rank-ordering of hits from screening (Figure 3A). Even at very low saturation times, which compensate for T_1_ relaxation effects of the ligands (Yan et al., 2003), a clear guideline for fragment ranking remains elusive (Figure S2).

Until now, all CORCEMA-ST calculations assumed a diffusion-limited on-rate kinetics of *k*_on_ = 10^9^ M^−1^ s^−1^ in line with previous calculations (Jayalakshmi and Krishna, 2002). However, the influence of kinetics on the correlation of STD amplification factor and fragment affinity became already apparent for PDE10A (Figure 2A). To systematically elucidate the consequences of slower binding kinetics on STD NMR screening of fragments, carbonic anhydrase II (CA2) was chosen as a model, as it is a well-studied example for which fast kinetics cannot be assumed. On-rates between 10^3^ and 10^6^ M^−1^ s^−1^ have been reported for low-molecular-weight inhibitors of CA2 (Navratilova and Hopkins, 2010). Therefore, we analyzed CA2 in complex with ethyl (2Z,4R)-2-(sulfamoylimino)-1,3-thiazolidine-4-carboxylate already included in previous data sets (PDB ID: 4FPT) varying the exchange kinetics (Figure 3B). If the on-rate is below the diffusion-limited threshold of 10^7^ M^−1^ s^−1^, the affinity range of fragments receiving high saturation transfer decreases. Consequently, low saturation transfer can be interpreted as originating either from high- or low-affinity fragments. Fragments with low micromolar affinity will give rise to similar STD effects as low millimolar binders. This potential caveat became more severe with slower on-rates. The same results from CORCEMA-ST calculations were observed when analyzing FAK1 and GSK3b kinases as well as increasing the molecular weight of these receptors (Figures S3, S4). Moreover, analyzing the same data, but highlighting the off-rates instead, emphasizes the off-rate bias in STD NMR screening (Figure 3C and Figure S4B). Oversimplified, one can state that a higher off-rate leads to increasing numbers of saturated ligand molecules and consequently to a higher STD effect. Hence, applying a rank-ordering based on STD effect during screening can lead to an accumulation of ligands with fast off-rate kinetics, contradicting current efforts to identify hits with slow off-rates (Copeland et al., 2006).

Since the underlying pathways leading to efficient saturation transfer from a target receptor to a ligand are multifactorial, it is difficult to identify a single determinant responsible for the lack of correlation between STD amplification factor and affinity. However, our results suggest that subtle changes in the binding site geometry and binding kinetics can already significantly alter the size of the STD amplification factor.

## Conclusion

In this study, we calculated STD NMR amplification factors for fragments identified in an experimental NMR screening against langerin (Aretz et al., 2018) and relate them to affinity. To expand these findings and rule out flaws in our analysis originating from experimental imperfections, we simulated different pairs of receptors and drug-like ligands using CORCEMA-ST. Varying saturation time, receptor size, binding kinetics, and interaction site in CORCEMA-ST simulations, there were no conditions in which the STD NMR amplification factor correlated unambiguously with affinity. These findings are in line with our experimental data. In conclusion, these data exemplify that assuming the observed STD effect relates to affinity and thereby allowing rank-ordering of hits from STD NMR fragment-based screening is misleading.

## Author Contributions

CR designed the study. JA performed experiments and statistical analysis. CR performed CORCEMA-ST calculations. Both authors contributed to manuscript writing and revision, and read and approved the submitted version.

### Conflict of Interest Statement

The authors declare that the research was conducted in the absence of any commercial or financial relationships that could be construed as a potential conflict of interest.

## References

[B1] Aretz J. Anumala U. R. Fuchsberger F. F. Molavi N. Ziebart N. Zhang H.. (2018). Allosteric inhibition of a mammalian lectin. J. Am. Chem. Soc. 140, 14915–14925. 10.1021/jacs.8b08644 30303367

[B2] Baker M. (2012). Fragment-based lead discovery grows up. Nat. Rev. Drug Discov. 12, 5–7. 10.1038/nrd3926 23274457

[B3] Barelier S. Eidam O. Fish I. Hollander J. Figaroa F. Nachane R.. (2014). Increasing chemical space coverage by combining empirical and computational fragment screens. ACS Chem. Biol. 9, 1528–1535. 10.1021/cb5001636 24807704 PMC4215856

[B4] Begley D. W. Moen S. O. Pierce P. G. Zartler E. R. (2013). Saturation transfer difference NMR for fragment screening. Curr. Protoc. Chem. Biol. 5, 251–268. 10.1002/9780470559277.ch130118 24391096

[B5] Bollag G. Hirth P. Tsai J. Zhang J. Ibrahim P. N. Cho H.. (2010). Clinical efficacy of a RAF inhibitor needs broad target blockade in BRAF-mutant melanoma. Nature 467, 596–599. 10.1038/nature09454 20823850 PMC2948082

[B6] Cala O. Krimm I. (2015). Ligand-orientation based fragment selection in STD NMR screening. J. Med. Chem. 58, 8739–8742. 10.1021/acs.jmedchem.5b01114 26492576

[B7] Chemical Computing Group ULC (2018). Molecular Operating Environment (MOE) (Montreal, QC).

[B8] Copeland R. A. Pompliano D. L. Meek T. D. (2006). Drug–target residence time and its implications for lead optimization. Nat. Rev. Drug Discov. 5, 730–739. 10.1038/nrd2082 16888652

[B9] Gossert A. D. Jahnke W. (2016). NMR in drug discovery: a practical guide to identification and validation of ligands interacting with biological macromolecules. Prog. Nucl. Magn. Reson. Spectrosc. 97, 82–125. 10.1016/j.pnmrs.2016.09.001 27888841

[B10] Hann M. M. Keserü G. M. (2012). Finding the sweet spot: the role of nature and nurture in medicinal chemistry. Nat. Rev. Drug Discov. 11, 355–365. 10.1038/nrd3701 22543468

[B11] Jayalakshmi V. Krishna N. R. (2002). Complete Relaxation and Conformational Exchange Matrix (CORCEMA) analysis of intermolecular saturation transfer effects in reversibly forming ligand–receptor complexes. J. Magn. Reson. 155, 106–118. 10.1006/jmre.2001.2499 11945039

[B12] Jayalakshmi V. Krishna N. R. (2005). Determination of the conformation of trimethoprim in the binding pocket of bovine dihydrofolate reductase from a STD-NMR intensity-restrained CORCEMA-ST optimization. J. Am. Chem. Soc. 127, 14080–14084. 10.1021/ja054192f 16201830

[B13] Jose R. A. Voet A. Broos K. Jakobi A. J. Bruylants G. Egle B.. (2012). An integrated fragment based screening approach for the discovery of small molecule modulators of the VWF–GPIbα interaction. Chem. Commun. 48, 11349–11351. 10.1039/c2cc35269a 23072895

[B14] Mayer M. Meyer B. (1999). Characterization of ligand binding by saturation transfer difference NMR spectroscopy. Angew. Chem. Int. Ed. 38, 1784–1788. 29711196 10.1002/(SICI)1521-3773(19990614)38:12<1784::AID-ANIE1784>3.0.CO;2-Q

[B15] Mayer M. Meyer B. (2001). Group epitope mapping by saturation transfer difference NMR to identify segments of a ligand in direct contact with a protein receptor. J. Am. Chem. Soc. 123, 6108–6117. 10.1021/ja0100120 11414845

[B16] Meyer B. Peters T. (2003). NMR spectroscopy techniques for screening and identifying ligand binding to protein receptors. Angew. Chem. Int. Ed. 42, 864–890. 10.1002/anie.200390233 12596167

[B17] Navratilova I. Hopkins A. L. (2010). Fragment screening by surface plasmon resonance. ACS Med. Chem. Lett. 1, 44–48. 10.1021/ml900002k 24900174 PMC4007845

[B18] Recht M. I. Sridhar V. Badger J. Bounaud P.-Y. Logan C. Chie-Leon B.. (2014). Identification and optimization of PDE10A inhibitors using fragment-based screening by nanocalorimetry and X-ray crystallography. J. Biomol. Screening 19, 497–507. 10.1177/1087057113516493 24375910 PMC4007584

[B19] Rees D. C. Congreve M. Murray C. W. Carr R. (2004). Fragment-based lead discovery. Nat. Rev. Drug Discov. 3, 660–672. 10.1038/nrd1467 15286733

[B20] Roughley S. D. Hubbard R. E. (2011). How well can fragments explore accessed chemical space? A case study from heat shock protein 90. J. Med. Chem. 54, 3989–4005. 10.1021/jm200350g 21561141

[B21] Souers A. J. Leverson J. D. Boghaert E. R. Ackler S. L. Catron N. D. Chen J.. (2013). ABT-199, a potent and selective BCL-2 inhibitor, achieves antitumor activity while sparing platelets. Nat. Med. 19, 202–208. 10.1038/nm.3048 23291630

[B22] Szczepina M. G. Bleile D. W. Pinto B. M. (2011). Investigation of the binding of a carbohydrate-mimetic peptide to its complementary anticarbohydrate antibody by STD-NMR spectroscopy and molecular-dynamics simulations. Chem. Eur. J. 17, 11446–11455. 10.1002/chem.201100222 21953925

[B23] Yan J. Kline A. D. Mo H. Shapiro M. J. Zartler E. R. (2003). The effect of relaxation on the epitope mapping by saturation transfer difference NMR. J. Magn. Reson. 163, 270–276. 10.1016/S1090-7807(03)00106-X 12914842

