# Supplementary material for: Ranking Hits From Saturation Transfer Difference Nuclear Magnetic Resonance–Based Fragment Screening
Source: Front Chem. 2019 Apr 12;7:215. doi: 10.3389/fchem.2019.00215 (PMC6473174; doi:10.3389/fchem.2019.00215)
Supplement: Supplementary file 1 [file Table_1.DOCX]

**Ranking hits from STD NMR-based fragment screening**

Jonas Aretz^1,2^, Christoph Rademacher^1,2^*

^1^Department of Biomolecular Systems, Max Planck Institute of Colloids and Interfaces, Research Campus Golm, Potsdam, Germany

^2^Department of Biology, Chemistry, and Pharmacy, Freie Universität Berlin
Takustraße 3, 14195 Berlin, Germany

**Contents**

| Figure S1: Influence of different saturation times. | 1 |
| --- | --- |
| Figure S2: Influence of the receptor size. | 2 |
| Figure S3: Influence of the on-rate of ligands binding to FAK1 kinase. | 2 |
| Figure S4: Influence of on- and off-rate of ligands binding to GSK3b kinase. | 3 |
| Figure S5: Evaluating the cut-off distance for CORCEMA-ST calculations. | 4 |
| Table S1: Kinetic data and crystal structures used for CORCEMA-ST calculations. | 5 |
| Table S2: Hits against murine langerin used for Figures 1a and 2e. | 8 |

**Additional data**

**
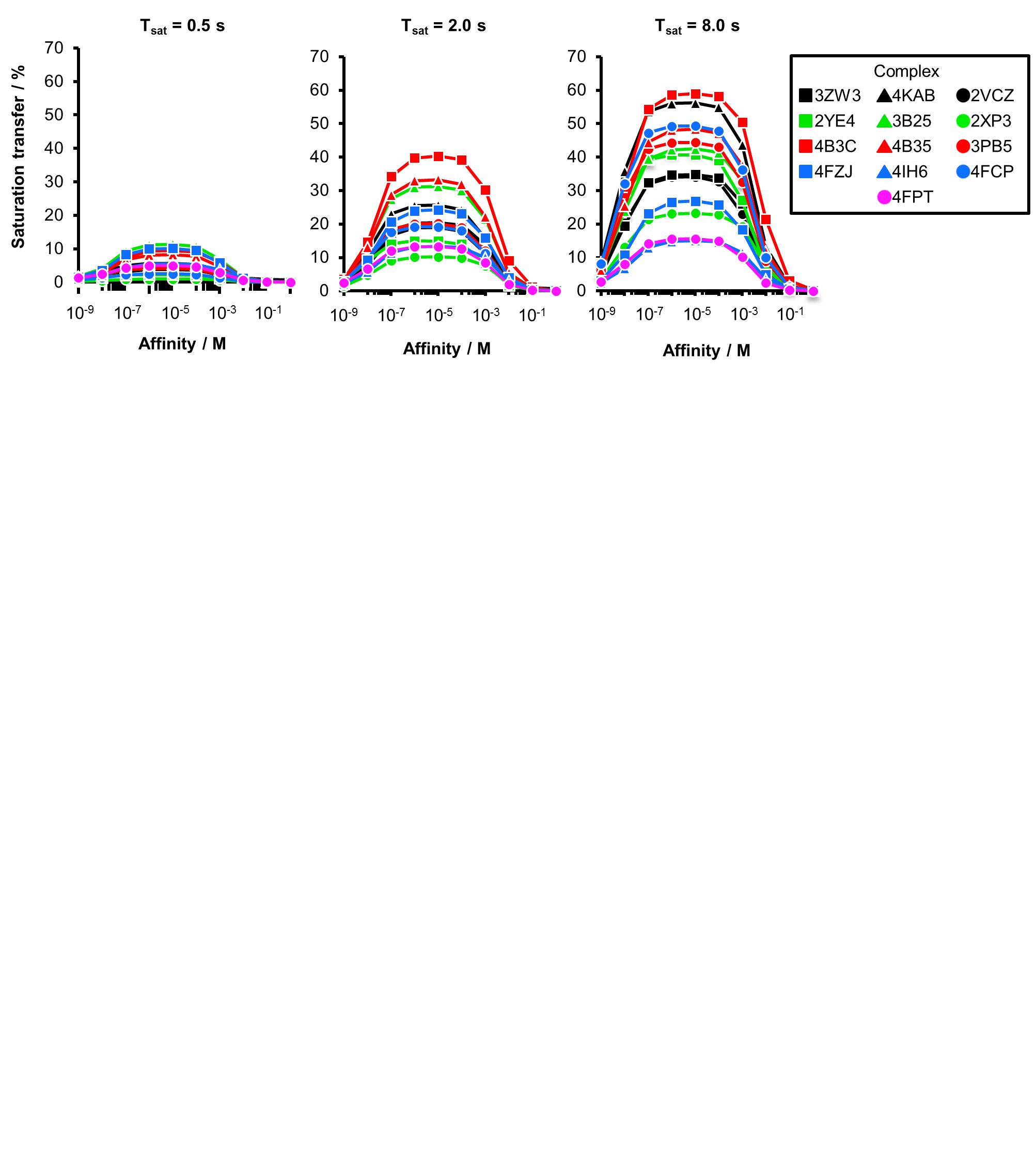
**

**Figure S1: Influence of different saturation times on the saturation transfer.**

Individual plots of 13 receptor-ligand complexes (Tab. 1) highlighting the dispersion of saturation transfer with varying saturation times for a broad range of affinities. For CORCEMA-ST calculations typical STD NMR screening conditions were assumed: [P] = 20 µM, [L] = 1.0 mM, saturation times from left to right are 0.5 s, 2.0 s, and 8.0 s, τ_c,bound_ = 30 ns (corresponding to 50 kDa molecular weight), and k_on_ = 10^9^ M^-1^ s^-1^.

**
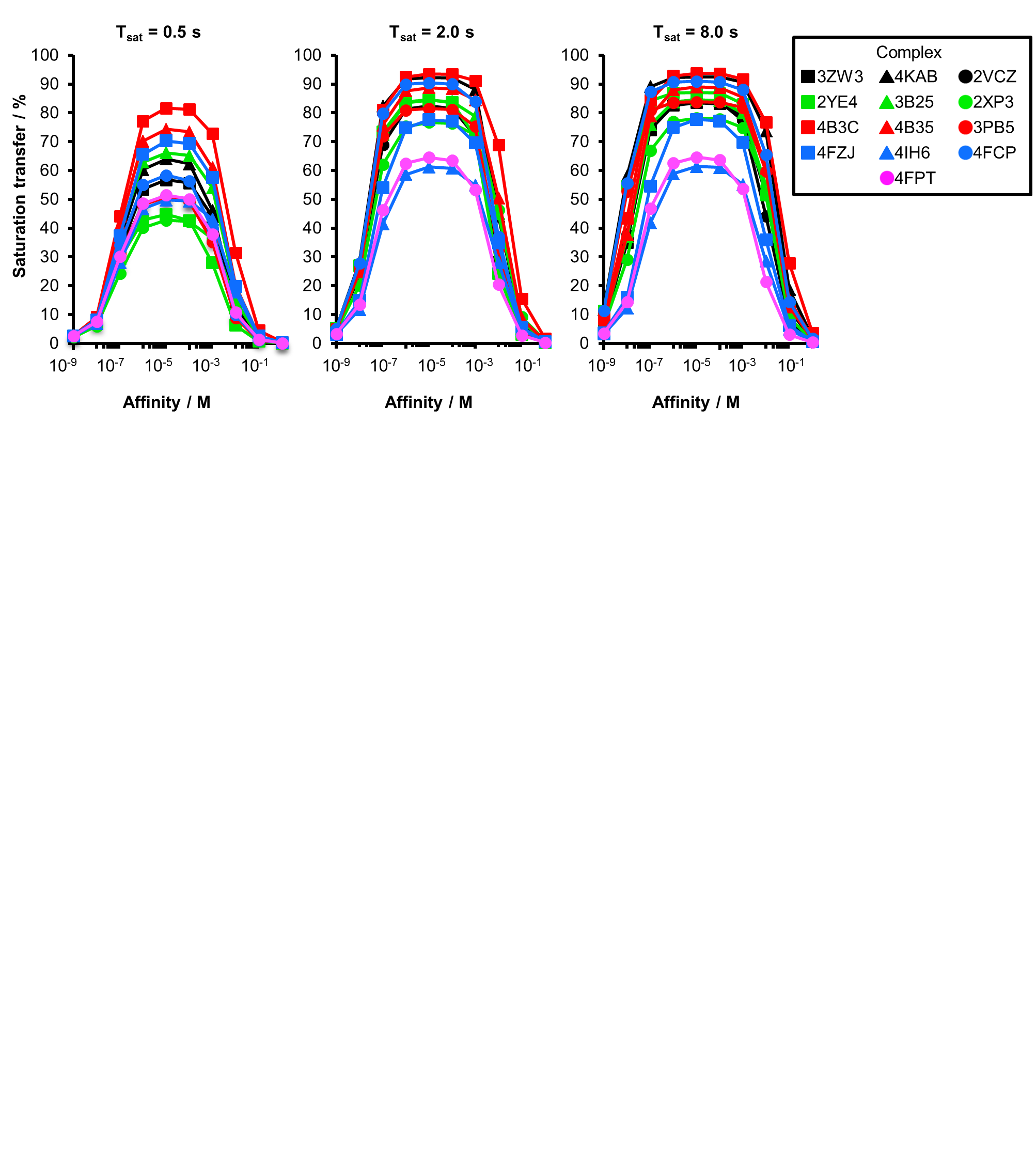
**

**Figure S2: Influence of different saturation times on the saturation transfer in a large receptor (166 kDa).**

Individual plots of 13 receptor-ligand complexes (Tab. 1) highlighting the dispersion of saturation transfer with varying saturation times of a large receptor for a broad range of affinities. For CORCEMA-ST calculations typical STD NMR screening conditions were assumed: [P] = 20 µM, [L] = 1.0 mM, saturation times = 0.5 s, 2.0 s, and 8.0 s, τ_c,bound_ = 100 ns (corresponding to 166 kDa molecular weight), and k_on_ = 10^9^ M^-1^ s^-1^.

**
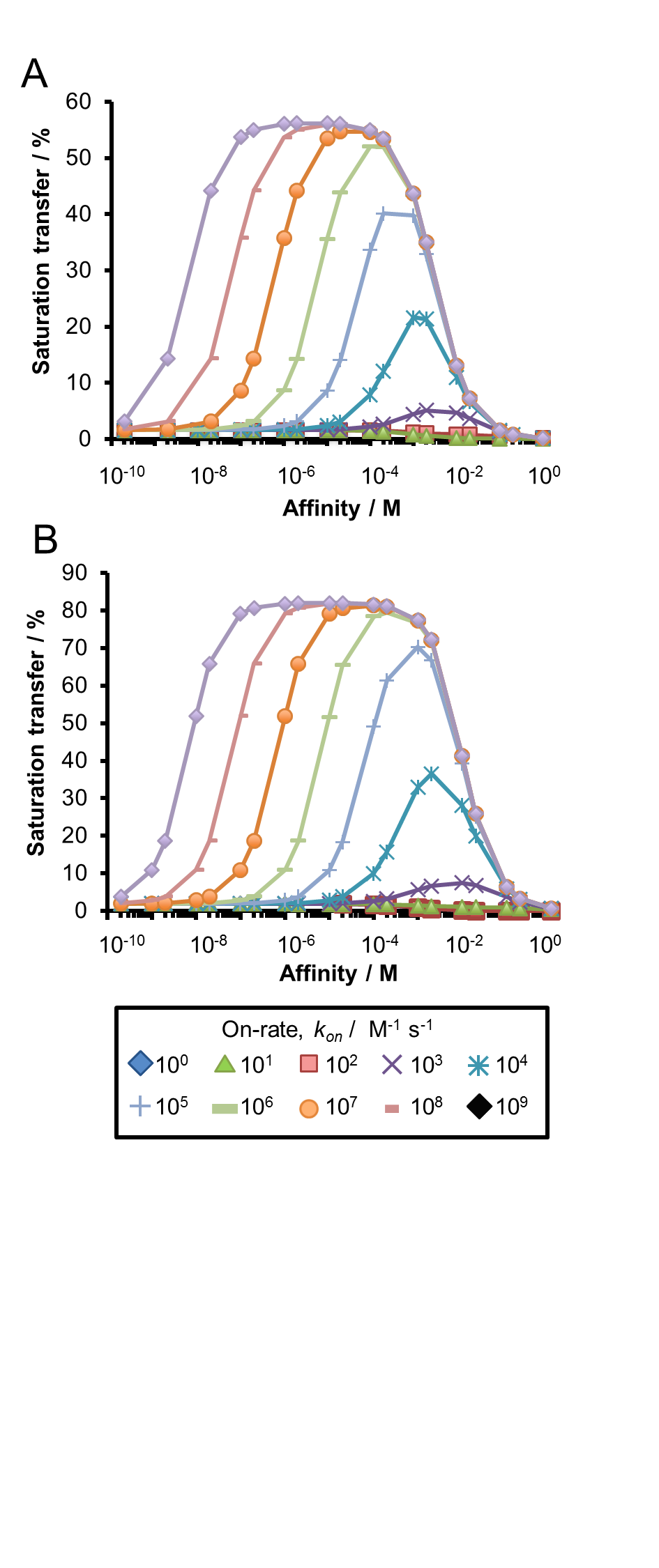
**

**Figure S3: Influence of the on-rate of ligands binding to FAK1 kinase.**

CORCEMA-ST calculations for FAK1 in complex with 3-methyl-1,5-dihydropyrazolo[4,3-c]pyrazole varying on-rates and correlation times of (A) τ_c_ = 30 ns and (B) τ_c_ = 100 ns of the receptor/ligand complex. Saturation times of 8.0 s are shown (PDB ID: 4KAB, [P] = 20 µM, [L] = 1.0 mM).


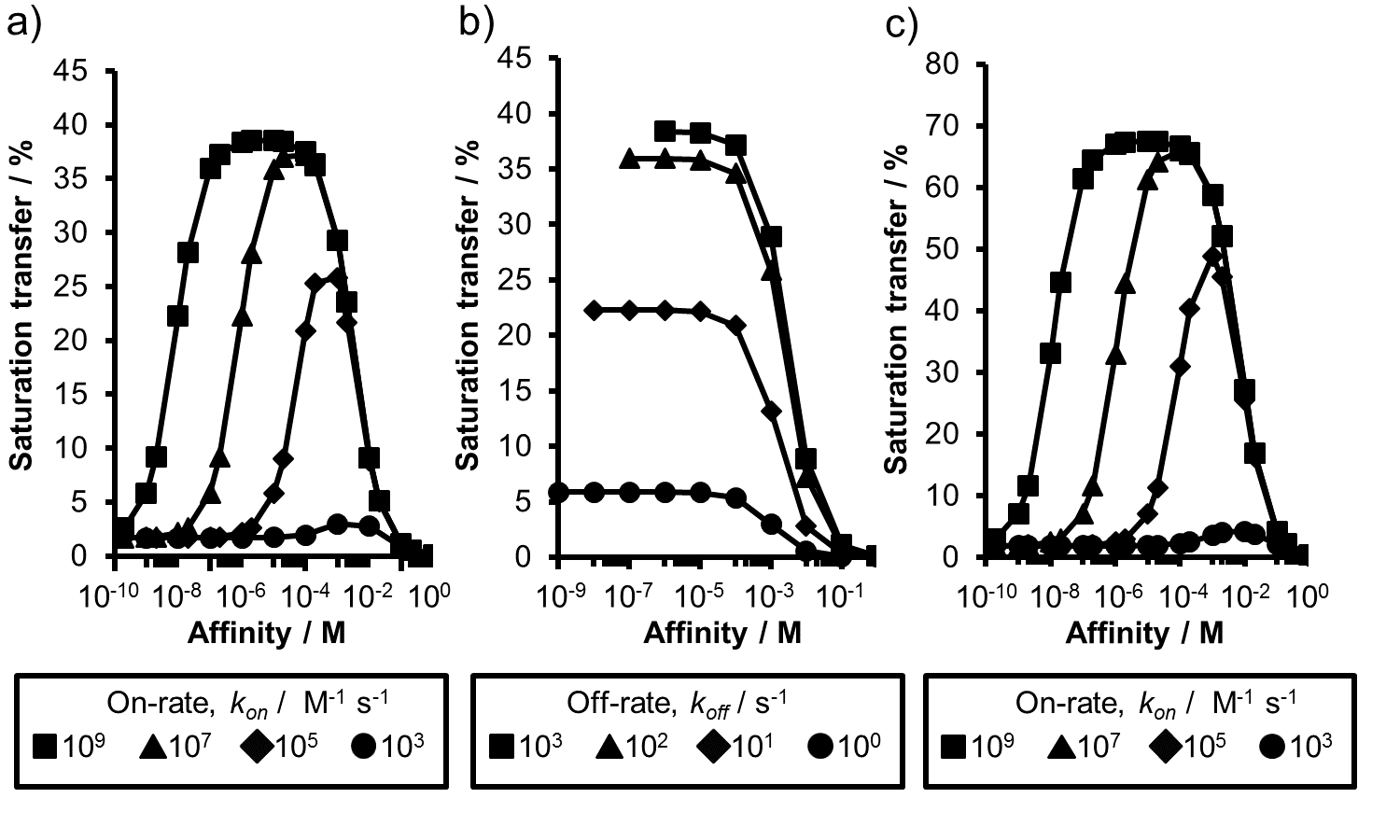


**Figure S4: Influence of on- and off-rate of ligands binding to GSK3b kinase.**

(A, B) CORCEMA-ST calculations for GSK3b kinase varying on- and off-rates of the receptor/ligand complex. Saturation times of 8.0 s are shown (PDB ID: 4J71, [P] = 20 µM, [L] = 1.0 mM). (C) CORCEMA-ST calculations for GSK3b kinase varying on-rates while using a correlation time of τ_c_ = 100 ns.


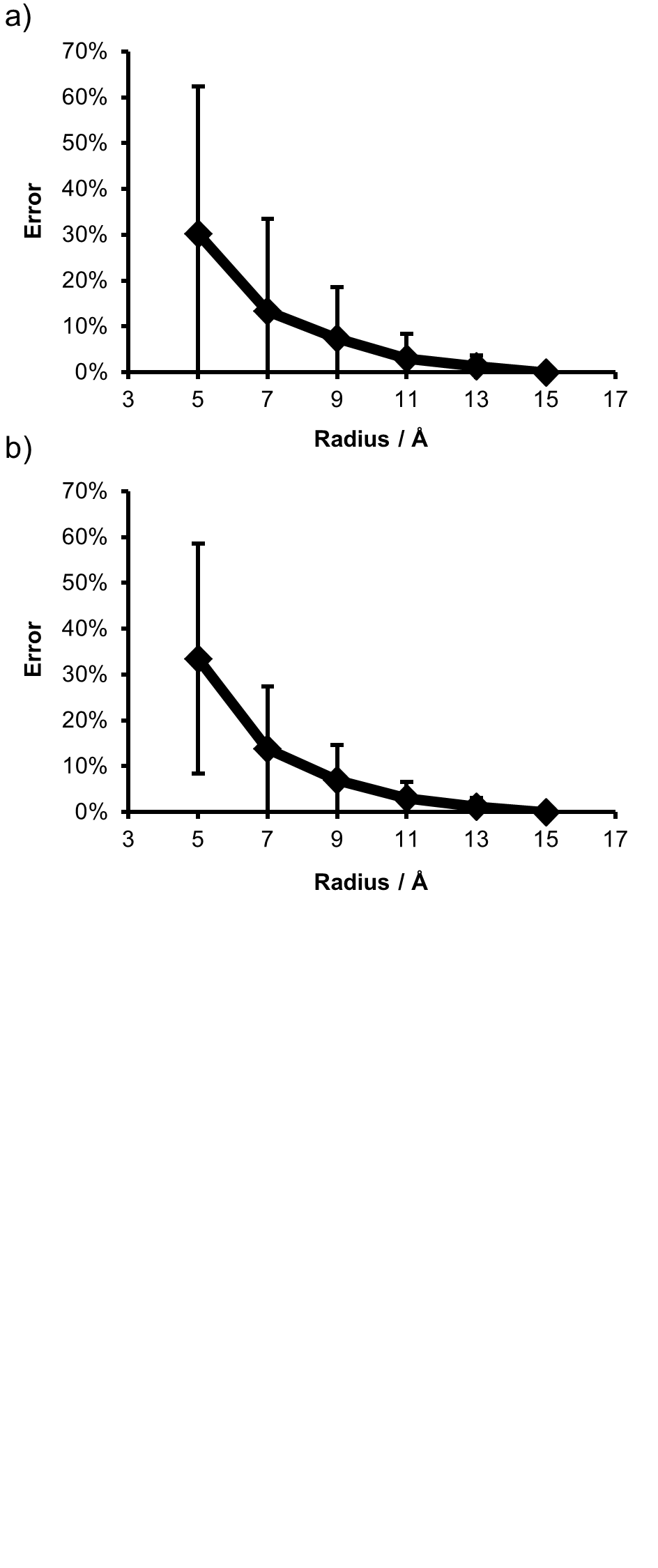


**Figure S5**: Evaluating the cut-off distance for CORCEMA-ST calculations for (a) 10 ns and (b) 100 ns correlation time of the receptor. Thirteen receptor-ligand complexes (Tab. 1) were used to calculate STD effects over a broad range of distances (from 5 to 15 Å) around each ligand hydrogen for which receptor protons were taken into account during the calculation. Assuming a large and computationally expensive cut-off of 15 Å would yield no errors originating from removing important hydrogens of the receptor’s relaxation network, the plot shows an increasing error with smaller distances for the cut-off. Consequently, for all CORCEMA-ST calculations 11 Å were used. The following settings were chosen for the calculations: [P] = 20 µM, [L] = 1.0 mM, saturation time = 2.0 s, and k_on_ = 10^9^ M^-1^ s^-1^

**Tables**

**Table S1: Kinetic data and crystal structures used for CORCEMA-ST calculations.**

| **Protein** | **Protein ID^a^** | **Compound** | **Compound ID^b^** | **PDB of complex** | **Affinity / µM** |
| --- | --- | --- | --- | --- | --- |
| **Figure 1, 3, S1, S2, S3** | | | | | |
| Peptidyl-prolyl cis-trans isomerase NIMA-interacting 1 | PIN1; Q13526 |  | CHEMBL372832 | 2XP3 |  |
| Heat shock protein HSP 90-alpha | HSP90AA1; P07900 |  | CHEMBL539379 | 2YE4 |  |
|  |  |  | CHEMBL1834091 | 3B25 |  |
|  |  |  | CHEMBL407391 | 4FCP |  |
| Endothiapepsin | EAPA; P11838 |  | CHEMBL1921971 | 3PB5 |  |
| DNA repair and recombination protein RadA | radA; O74036 |  | CHEMBL404923 | 4B3C |  |
|  |  |  | CHEMBL3416133 | 4B35 |  |
| Pantothenate synthetase | panC; P9WIL5 |  | - | 4FZJ |  |
| HCV nonstructural protein 5B | NS5B; P26663 |  | CHEMBL2324309 | 4IH6 |  |
| Carbonic anhydrase 2 | CA2; P00918 |  | - | 4FPT |  |
| Hematopoietic prostaglandin D synthase | HPGDS; O60760 |  | CHEMBL264950 | 2VCZ |  |
| Phosphatidylinositol 4,5-bisphosphate 3-kinase catalytic subunit gamma isoform | PIK3CG; P48736 |  | CHEMBL1914475 | 3ZW3 |  |
| Focal adhesion kinase 1 | PTK2; Q05397 |  | CHEMBL1599242 | 4KAB |  |
| **Figure 2** | | | | | |
| cAMP and cAMP-inhibited cGMP 3',5'-cyclic phosphodiesterase 10A | PDE10A; Q9Y233 | 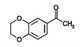 | - | 4LM3 | 1200 |
|  |  | 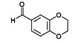 | - | 4LM2 | 940 |
|  |  | 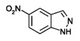 | CHEMBL165372 | 4LM0 | 560 |
|  |  | 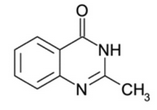 | CHEMBL395092 | 4LLK | 990 |
| Beta-lactamase | ampC; P00811 | 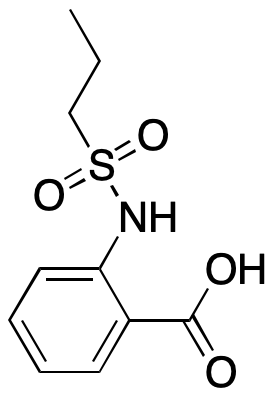 | - | 4KZ4 | 70 |
|  |  | 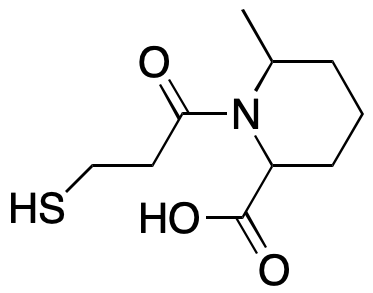 | CHEMBL331575 | 4KZ6 | 800 |
|  |  | 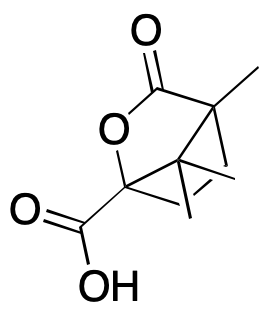 | - | 4KZ7 | 3200 |
|  |  | 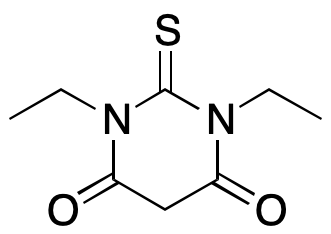 | CHEMBL2136061 | 4KZ8 | 1600 |
|  |  | 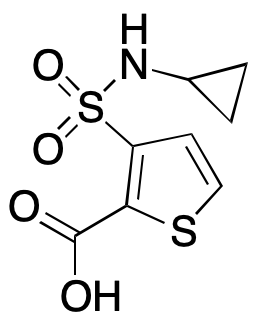 | - | 4KZA | 200 |
|  |  | 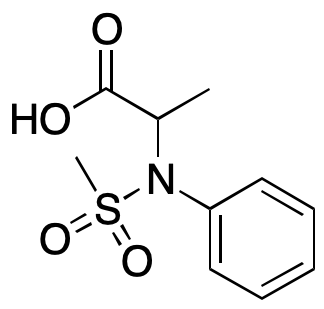 | - | 4KZB | 1300 |
| Heat shock protein HSP 90-alpha | HSP90AA1; P07900 |  | CHEMBL540934 | 2YE2 | 490 |
|  |  |  | CHEMBL539379 | 2YE4 | 570 |
|  |  |  | CHEMBL1738712 | 2YE5 |  |
|  |  |  | CHEMBL43175 | 2YE6 | >400 |
|  |  |  | - | 2YE7 |  |
|  |  |  | - | 2YEA |  |
|  |  |  | - | 2YEB |  |
|  |  |  | - | 2YEC |  |
|  |  |  | CHEMBL226345 | 2YED |  |
|  |  |  | CHEMBL304009 | 2YEE |  |
|  |  |  | CHEMBL1178 | 2YEG |  |
|  |  |  | CHEMBL1834091 | 3B25 |  |
|  |  |  | CHEMBL407391 | 4FCP |  |
| **Figure S4** | | | | | |
| Glycogen synthase kinase-3 beta | GSK3B; P49841 |  | CHEMBL3941552 | 4J71 | >3000 |

a: Gene name; UniProt ID

b: CHEMBL ID (if available)

**Tab. S2: Hits against murine langerin (Cd207; Q8VBC4) used for Figures 1a and 2e.**

| ID^a^ | Compound | Affinity / mM^b^ | STD amplification factor / - |
| --- | --- | --- | --- |
| **Figure 1a:** | | | |
| **1** | 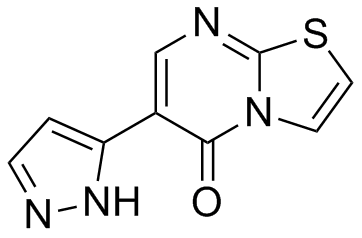 | 0.8 ± 0.1 | 16 |
| **S3** | 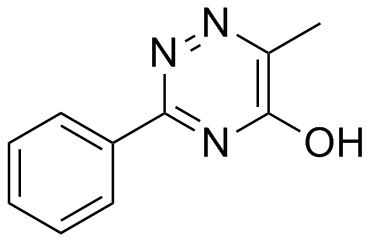 | 4.7 ± 0.6 | 19 |
| **S6** | 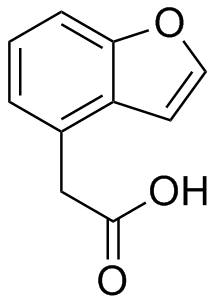 | 4.3 ± 0.5 | 55 |
| **S8** | 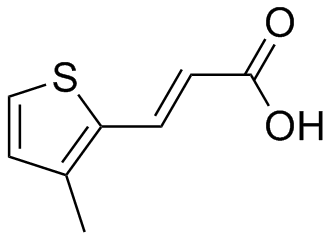 | 3.4 ± 0.4 | 86 |
| **S9** | 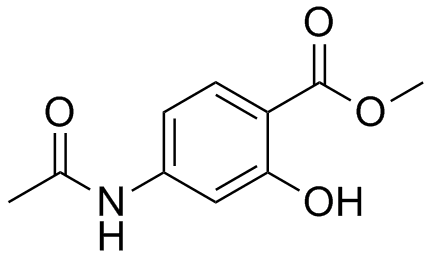 | 4.1 ± 0.9 | 31 |
| **S11** | 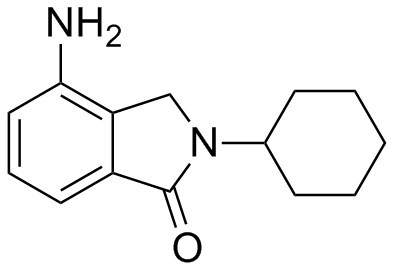 | 0.7 ± 0.2 | 23 |
| **S12** | 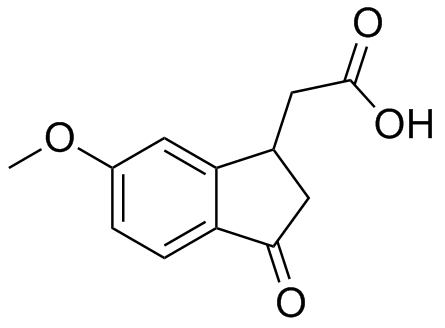 | 1.1 ± 0.2 | 10 |
| **S13** | 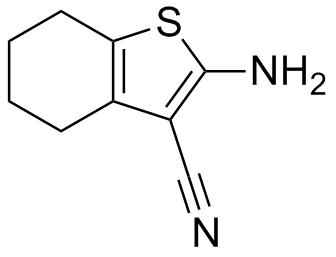 | 1.5 ± 0.3 | 33 |
| **S14** | 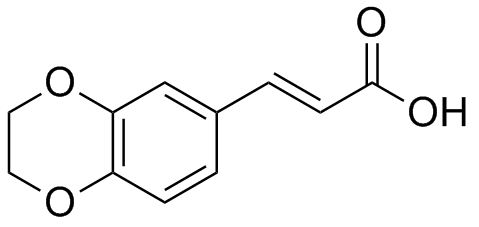 | 2.2 ± 0.2 | 36 |
| **S15** | 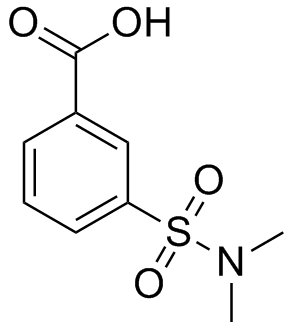 | 11.3 ± 3.0 | 41 |
| **S18** | 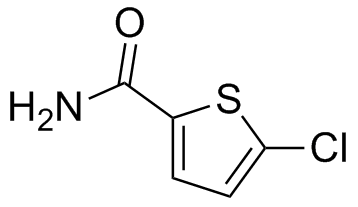 | 3.5 ± 0.2 | 21 |
| **S21** | 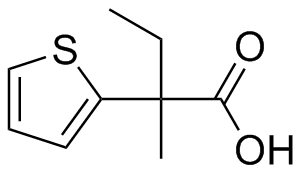 | 3.3 ± 0.2 | 13 |
| **S22** | 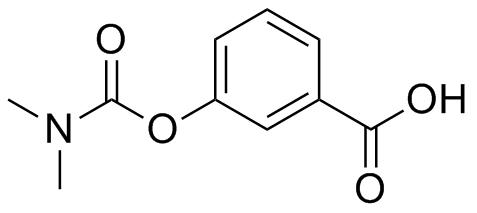 | ~4 | 24 |
| **S23** | 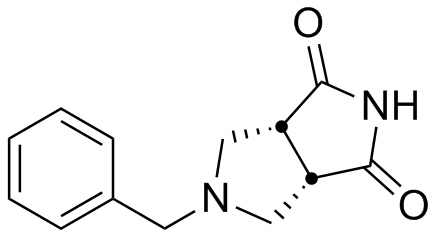 | 4.9 ± 0.2 | 4 |
| **S30** | 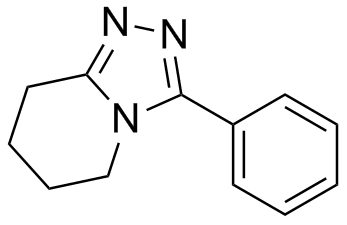 | 10.0 ± 3.0 | 8 |
| **S31** | 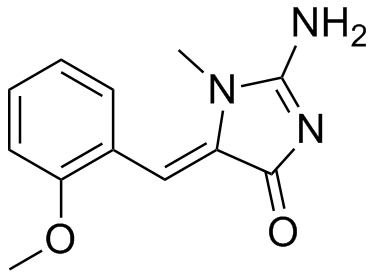 | 4.0 ± 2.0 | 5 |
| **S36** | 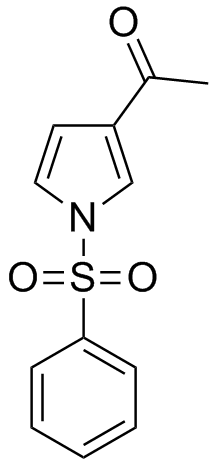 | 19.0 ± 1.0 | 44 |
| **S37** | 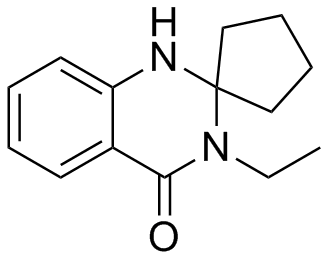 | 13.0 ± 2.0 | 2 |
| **S38** | 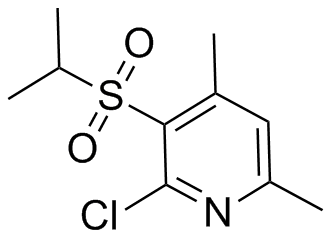 | 4.7 ± 0.7 | 4 |
| **S39** | 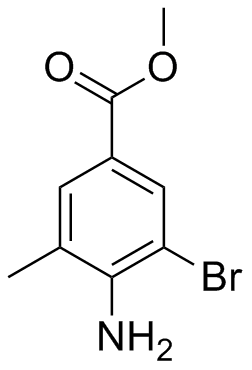 | 28.0 ± 9.0 | 49 |
| **S42** | 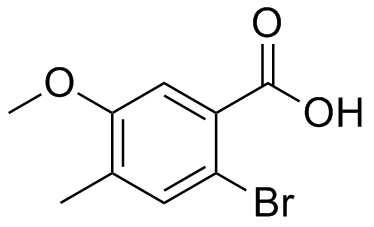 | 5.4 ± 0.9 | 79 |
| **S44** | 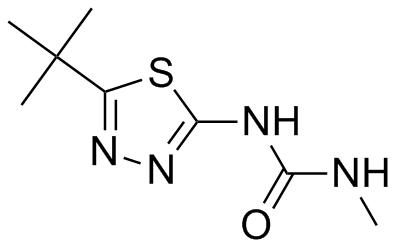 | 2.9 ± 0.4 | 7 |
| **S52** | 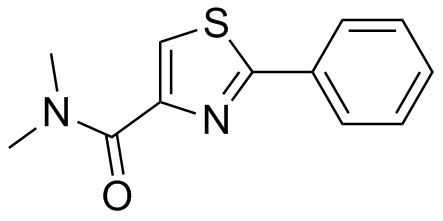 | 2.7 ± 0.4 | 3 |
| **S53** | 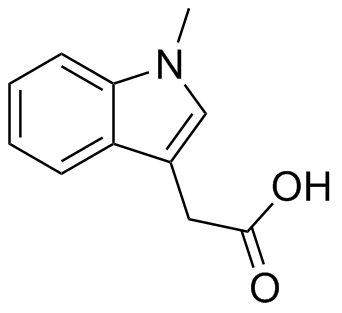 | 3.0 ± 0.1 | 37 |
| **S55** | 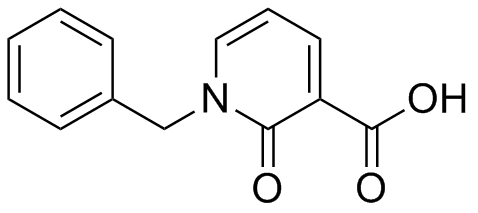 | 24.7 ± 10.3 | 10 |
| **S56** | 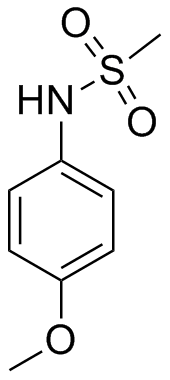 | 16.8 ± 3.6 | 3 |
| **S57** | 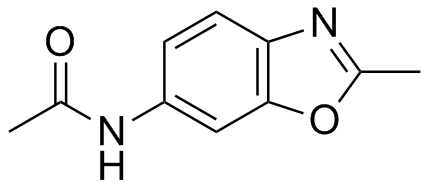 | 5.7 ± 0.4 | 2 |
| **S58** | 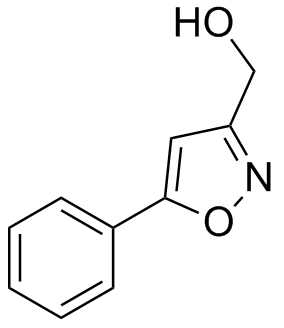 | 2.3 ± 0.1 | 19 |
| **S59** | 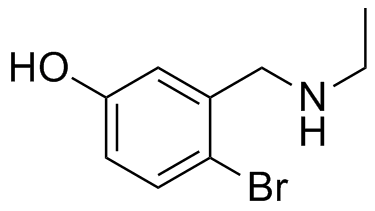 | 2.0 ± 0.3 | 13 |
| **S60** | 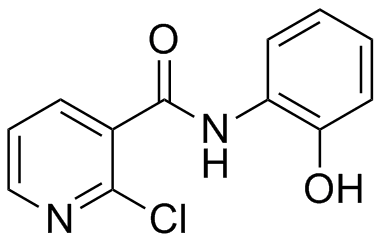 | 2.4 ± 0.1 | 24 |
| **S61** | 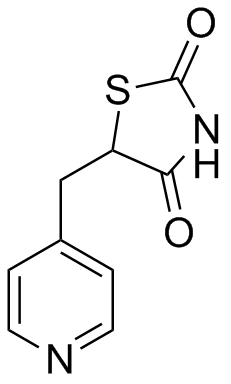 | 3.8 ± 0.3 | 16 |
| **S63** | 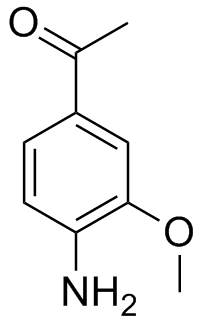 | 9.0 ± 1.0 | 4 |
| **S65** | 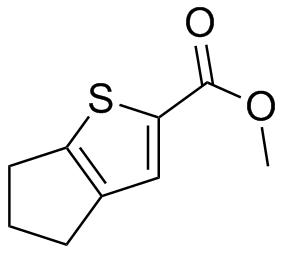 | 4.5 ± 0.7 | 43 |
| **S66** | 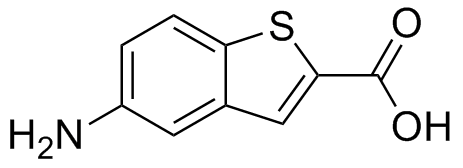 | 11.6 ± 0.1 | 44 |
| **Figure 2e:**  **** | | | |
| **1** | 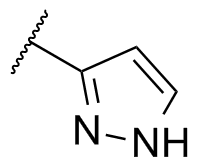 | 0.8 ± 0.1 | 1.8 |
| **4** | COOH | 0.8 ± 0.1 | 0.8 |
| **30** | CH3 | 3.2 ± 0.4 | 0.4 |
| **31** | 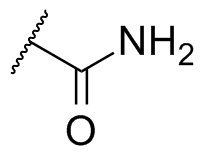 | 1.6 ± 1.0 | 0.7 |
| **33** | 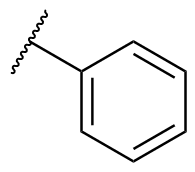 | 1.0 ± 0.1 | 4.7 |
| **35** | 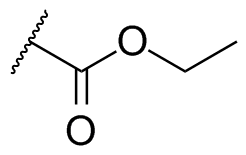 | 2.7 ± 0.2 | 1.2 |
| **36** | 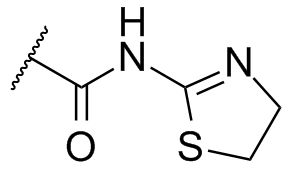 | 1.1 ± 0.1 | 2.2 |
| **37** | 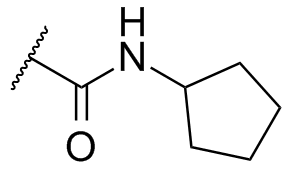 | 0.7 ± 0.1 | 2.0 |
| **50** | 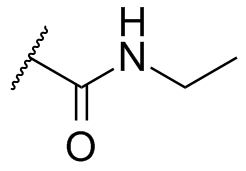 | 3.5 ± 0.4 | 0.8 |
| **51** | 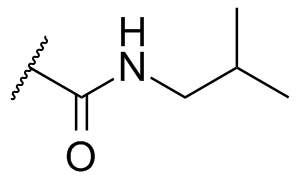 | 3.0 ± 0.5 | 4.2 |
| **55** | 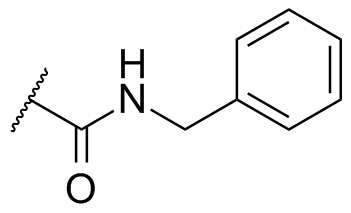 | 0.5 ± 0.1 | 1.3 |
| **78** | 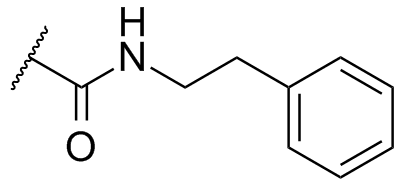 | 0.6 ± 0.1 | 0.8 |
| **91** | 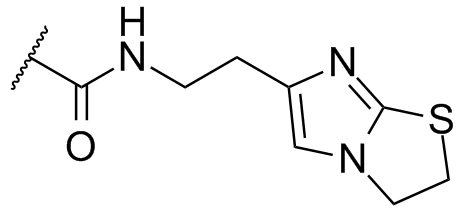 | 0.4 ± 0.1 | 1.6 |
| **94** | 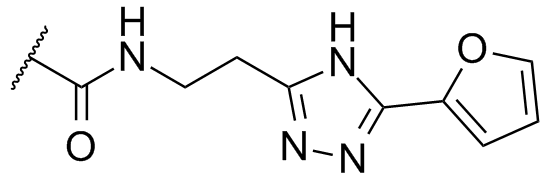 | 1.4 ± 0.1 | 2.9 |
| **95** | 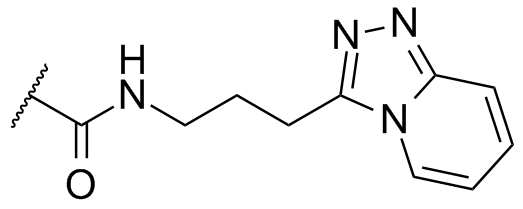 | 5.0 ± 0.7 | 2.1 |
| **111** | 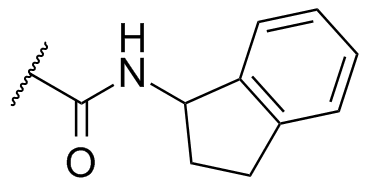 | 0.6 ± 0.2 | 4.2 |
| **124** | 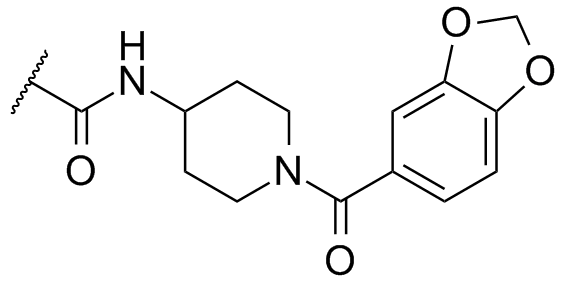 | 1.2 ± 0.1 | 4.0 |
| **125** | 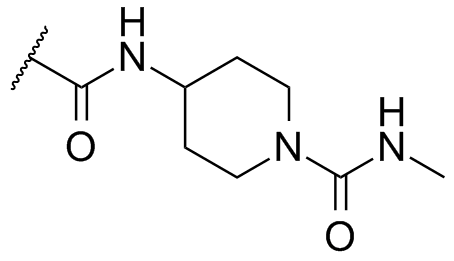 | 6.1 ± 0.4 | 0.8 |
| **127** | 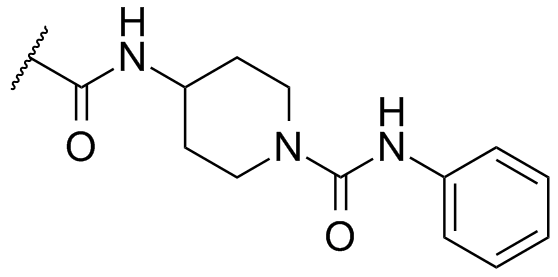 | 0.8 ± 0.1 | 4.3 |
| **128** | 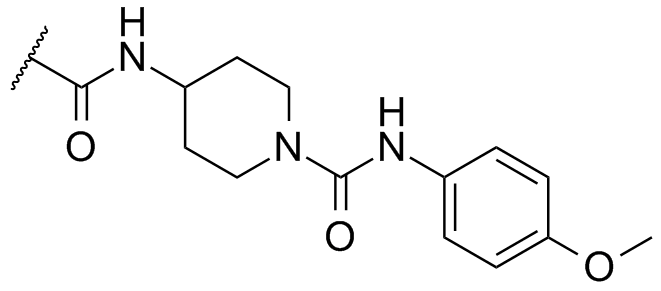 | 0.9 ± 0.1 | 5.4 |
| **134** | 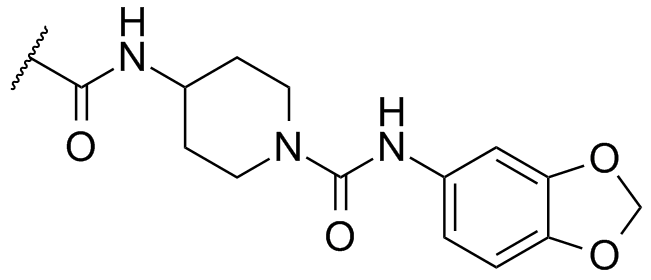 | 0.6 ± 0.1 | 6.3 |
| **135** | 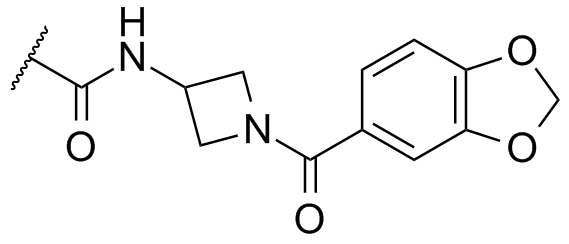 | 1.1 ± 0.3 | 4.9 |
| **140** | 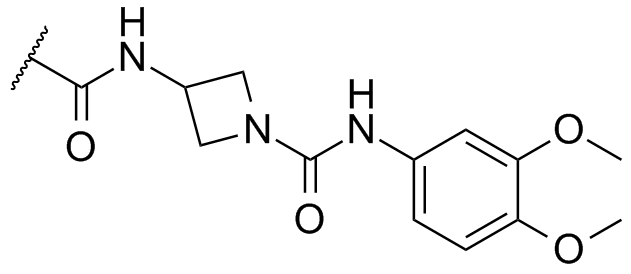 | 1.7 ± 0.1 | 5.0 |
| **141** | 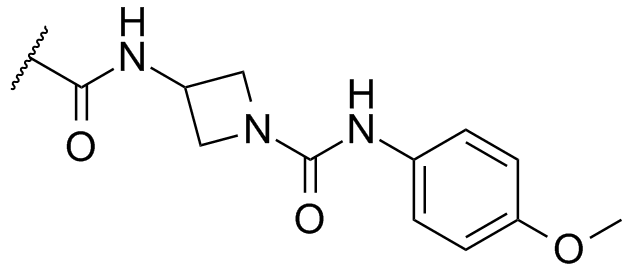 | 1.0 ± 0.1 | 1.2 |
| **142** | 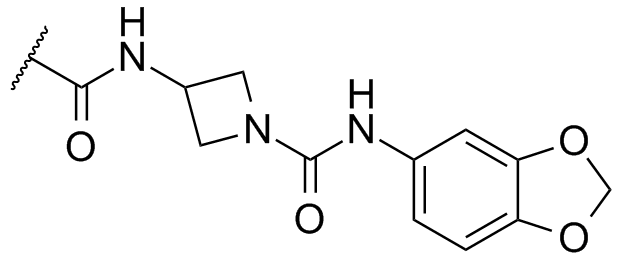 | 0.5 ± 0.1 | 1.7 |
| **147** | 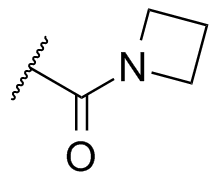 | 5.4 ± 0.7 | 0.2 |
| **153** | 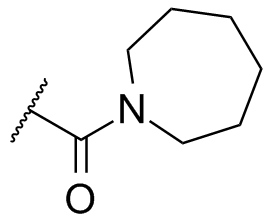 | 4.0 ± 0.3 | 0.8 |
| **154** | 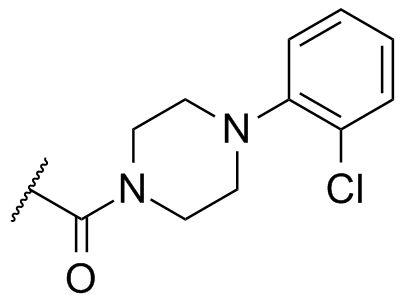 | 0.4 ± 0.1 | 4.1 |
| **155** | 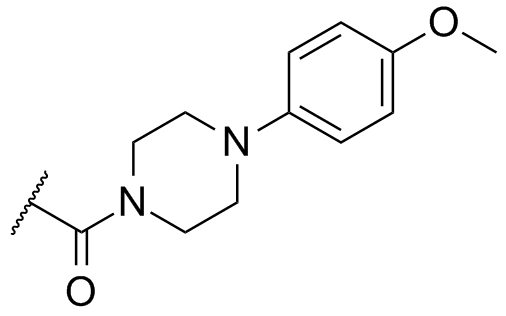 | 1.3 ± 0.2 | 2.5 |
| **157** | 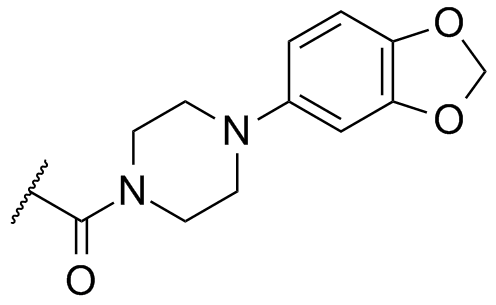 | 1.7 ± 0.1 | 1.7 |

^a^: Identifier used in Aretz *et al*. (2018).

^b^: Affinity estimated by SPR in Aretz *et al*. (2018).
